# Distinct impacts of tropical North Atlantic warming flavors on cross-basin tropical cyclone activity

**DOI:** 10.1126/sciadv.aeb2316

**Published:** 2026-05-20

**Authors:** Jiuwei Zhao, Luyao Bai, Ruifen Zhan, Yi Liu, Wenju Cai, Yuqing Wang, Jong-Seong Kug, Il-Ju Moon, Xiangbo Feng, Ralf Toumi, Botao Zhou, Leying Zhang, Han-Ching Chen

**Affiliations:** ^1^ State Key Laboratory of Climate System Prediction and Risk Management/Key Laboratory of Meteorological Disaster, Ministry of Education/Collaborative Innovation Center on Forecast and Evaluation of Meteorological Disasters, Nanjing University of Information Science and Technology, Nanjing, China.; ^2^ College of Ecology and Environment, Joint Innovation Center for Modern Forestry Studies, Nanjing Forestry University, Nanjing, China.; ^3^ Department of Atmospheric and Oceanic Sciences/Institute of Atmospheric Sciences, Fudan University, CMA-FDU Joint Laboratory of Marine Meteorology, Shanghai, China.; ^4^ Center for Ocean Research in Hong Kong and Macau/Department of Ocean Science, The Hong Kong University of Science and Technology, Hong Kong, China.; ^5^ Laoshan Laboratory, Qingdao, China.; ^6^ Frontiers Science Center for Deep Ocean Multispheres and Earth System and Key Laboratory of Physical Oceanography, Ocean University of China, Qingdao, China.; ^7^ State Key Laboratory of Marine Environmental Science and College of Ocean and Earth Sciences, Xiamen University, Xiamen, China.; ^8^ State Key Laboratory of Loess Science, Institute of Earth Environment, Chinese Academy of Sciences, Xi’an, China.; ^9^ Department of Atmospheric Sciences and International Pacific Research Center, School of Ocean and Earth Science and Technology, University of Hawaii at Manoa, Honolulu, HI 96822, USA.; ^10^ School of Earth and Environmental Sciences, Seoul National University, Seoul, South Korea.; ^11^ Typhoon Research Center, Jeju National University, Korea Typhoon Research Institute Corp., Jeju, South Korea.; ^12^ National Centre for Atmospheric Science and Department of Meteorology, University of Reading, Reading, UK.; ^13^ Department of Physics, Imperial College London, London, UK.

## Abstract

Tropical North Atlantic (TNA) warming typically favors tropical cyclone (TC) genesis over the North Atlantic but suppresses TC formation over the Northwest Pacific during boreal summer. The TNA anomaly patterns can be classified into an eastern coastal and a western warm-pool type, but their respective impacts remain unclear. Here, we find a pronounced difference in the impact between the two TNA flavors. The warm-pool TNA warming suppresses Northwest Pacific TC genesis through a remote dynamical control, while the coastal warming promotes North Atlantic TC genesis via a local thermodynamic control. High-resolution modeling reveals that, compared with the canonical TNA warming, the warm-pool TNA warming suppresses Northwest Pacific TC genesis by 65.2%, while the coastal warming enhances North Atlantic TC genesis by 60.1%. Under greenhouse warming, increased coastal TNA warming is projected to intensify North Atlantic TC activity. Therefore, distinguishing TNA flavors is critical for improving seasonal prediction and future projections of cross-basin TC activity.

## Teaser

Two Atlantic warming patterns have distinct impacts: Coastal boosts Atlantic cyclones and warm pool reduces Pacific typhoons.

## INTRODUCTION

Variability of sea surface temperature (SST) over the tropical North Atlantic (TNA) exerts both local and remote impacts, thereby modulating pan-tropical climate (*1*–*8*). In particular, warm TNA SST anomalies during a typical hurricane season (from June to November) substantially influence tropical cyclone (TC) activity in the North Atlantic and Northwest Pacific, which account for about 45% of global TCs. In the North Atlantic, a TNA warming enhances TC activity (fig. S1, A and B) by weakening the vertical wind shear, increasing mid-level moisture and low-level vorticity (*9*–*12*). In contrast, over the Northwest Pacific, the TNA warming suppresses TC genesis (fig. S1, A and C) via two distinct pathways. One involves a westward-propagating Rossby wave response, through which TNA warming modulates the eastern Pacific and subsequently triggers the wind-evaporation-SST feedback (*4*, *9*, *13*–*16*), and the other involves an eastward-propagating Kelvin wave response, wherein TNA warming alters Indian Ocean SSTs and affects the Northwest Pacific via an Indo-Pacific coupling (*15*, *17*, *18*). Given the socioeconomic vulnerability of densely populated coastal areas in the North Atlantic and Northwest Pacific, understanding how TNA warming modulates cross-basin TC activity is crucial for improving TC prediction and reducing associated risks.

**Fig. 1.**
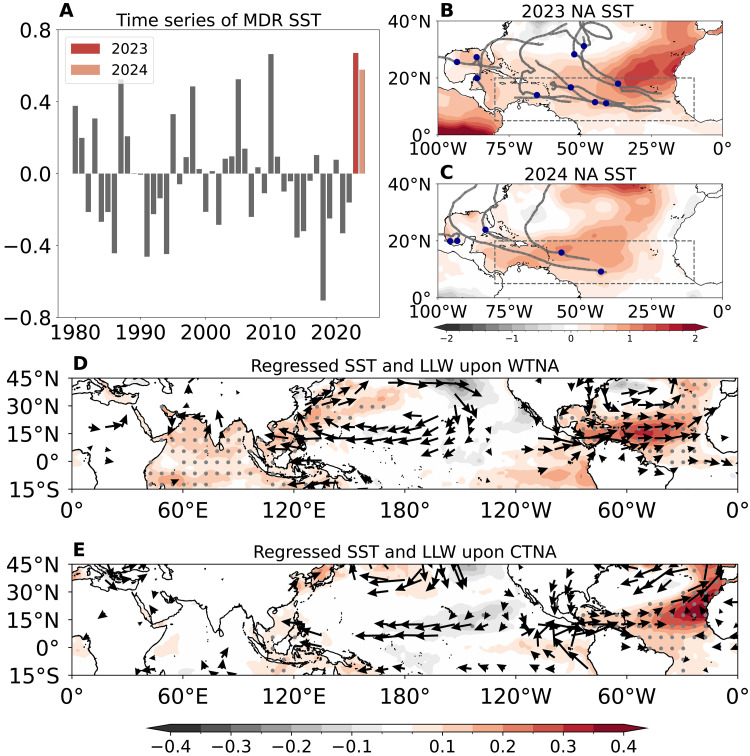
Contrasting patterns of TNA warming and associated large-scale circulation responses. (**A**) Time series of SST anomalies averaged over the main TC development region (MDR) over the North Atlantic (10°W to 80°W, 5°N to 20°N) during the hurricane season (June to November); (**B**) summer (June to August) North Atlantic (NA) SST anomalies in 2023 with the climatological mean and linear trend for 1980–2024 removed; (**C**) same as (B) but for 2024; (**D**) regressed SST anomalies (shading; in degrees Celsius) and low-level winds (LLWs) at 850 hPa (vector; in meters per second) during the hurricane season upon the WTNA index for 1980–2024 period; and (**E**) same as (D) but regressed upon the CTNA index. The SST anomalies in 2023 and 2024 are highlighted in dark and shallow red in (A). TC tracks and genesis locations are marked with black lines and blue dots in (B) and (C). The dots and black vectors in (D) and (E) represent areas where anomalies are statistically significant above the 90% confidence level based on the two-tailed Student’s *t* test.

The 2023 and 2024 witnessed consecutive extreme TNA warming during boreal summer and autumn (*9*, *10*, *19*–*21*). The main TC development region (MDR) (*21*, *22*), where a majority of North Atlantic TCs form, recorded very high SSTs in June to August of 2023 and 2024 (Fig. 1A). Despite a similarly warm Atlantic, North Atlantic TC activity exhibited a stark contrast in the 2 years: an active 2023 summer season (Fig. 1B) with 10 TCs generated from June to August versus a near-neutral 2024 summer season with only 5 TCs (Fig. 1C). This contrast is not readily attributable to El Niño–Southern Oscillation (ENSO), as the two summers occurred during the developing phases of El Niño in 2023 and La Niña in 2024—opposite phases that typically suppress and enhance North Atlantic TC genesis, respectively (*23*). Notably, most state-of-the-art models successfully predicted the active 2023 summer season but failed to predict the neutral 2024 summer season under similarly warm Atlantic conditions (*19*, *21*). These unusual features suggest that the TNA warming may have exerted different impacts on North Atlantic TC activity between 2023 and 2024.

Despite comparable magnitudes of TNA warming in 2023 and 2024 (Fig. 1A; 0.67°C versus 0.58°C), their anomaly patterns exhibit distinct spatial patterns, with the warming off the coastal region of Northwestern Africa in 2023 but over the Atlantic warm pool in 2024 (Fig. 1, B and C) (*20*). Such discrepancy raises a fundamental question as to whether different spatial patterns of TNA SST anomalies exert distinct impacts on TC activity. Here, we show that the two flavors of TNA SST anomalies are responsible for different impacts on cross-basin TC activity in the North Atlantic and the Northwest Pacific, underpinned by different dynamic and thermodynamic controls. Such different impacts are reproduced and quantified by high-resolution numerical experiments, with strong implications on prediction skills of Pacific-Atlantic TC activity. Climate model projection suggests that the 2023-like active TC season over the North Atlantic will likely become more frequent under greenhouse warming, owing to more frequent coastal TNA warming events resembling that of the 2023 episode.

## RESULTS

### TNA flavors and their distinct impacts on cross-basin TC genesis

To disentangle two TNA flavors, we apply a rotated empirical orthogonal function analysis of June to November SST anomalies from 1980 to 2024 (see the “Definitions of TNA, WTNA, and CTNA indices” section in Materials and Methods; fig. S2) and extract two distinct warm anomalous SST patterns, a warm-pool type (WTNA; Fig. 1D) and a coastal type (CTNA; Fig. 1E). The 2023 TNA warming featured a CTNA pattern with the SST anomaly centered off the Northwestern African coast, whereas the 2024 TNA warming showed a WTNA pattern with the SST anomaly center superimposed over the Atlantic warm pool (Fig. 1, B and C).

The WTNA warming resembles the canonical TNA warming pattern but exhibits weaker and vertically shallower wind anomalies (Fig. 1D versus fig. S1A). The WTNA triggers broad westerly wind anomalies, lacking a well-defined low-level cyclonic circulation (Fig. 1D), indicating a weaker coupling between TNA SST warming and low-level atmospheric vorticity. In contrast, the CTNA warming features warming confined to the Northwestern African coast without substantial Indian Ocean warming (Fig. 1E) (*15*, *17*, *18*), which excites a pronounced cyclonic circulation over the TNA (Fig. 1E), favoring enhanced TC genesis locally.

The situation is reversed over the Northwest Pacific. The WTNA warming triggers an anomalous low-level anticyclonic circulation (*24*–*26*), which suppresses TC genesis over the Northwest Pacific. This anomalous anticyclone is dynamically linked to upper-level Kelvin waves in response to Indian Ocean SST anomalies (fig. S3, A and B) (*15*, *17*, *18*, *24*–*27*). At low levels, the signal from Africa to the Indian Ocean is weak and statistically insignificant due to strong land surface damping (Fig. 1, D and E) (*27*). Conversely, the CTNA warming triggers only equatorial easterly anomalies in the eastern Northwest Pacific without an anticyclonic response in the Pacific (Fig. 1, D versus E), likely because the CTNA SST anomalies fail to initiate a Kelvin wave (fig. S3, B versus C), implying a weak teleconnection to the Indian Ocean (*27*). Instead, the CTNA modulates zonal SST gradients across the tropical Atlantic (Fig. 1, C and E), weakening the trade wind which consequently exerts a limited influence on Northwest Pacific TC genesis (*28*).

**Fig. 2.**
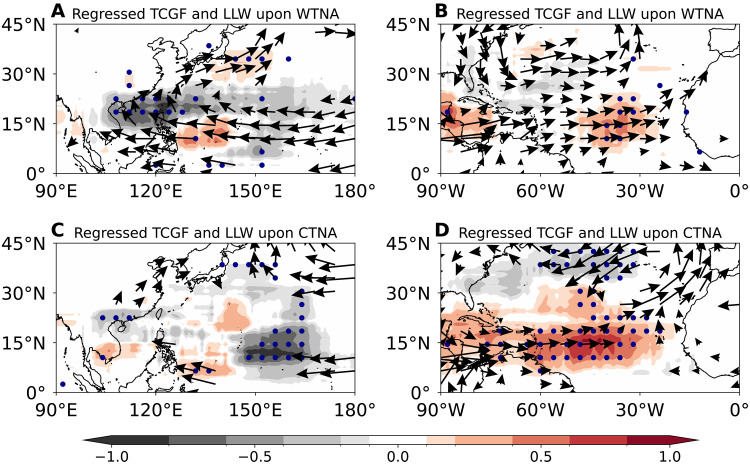
Impacts of the two flavors of TNA warming on TCGF and LLWs over the Northwest Pacific and the North Atlantic. (**A**) Regressed Northwest Pacific TCGF anomalies (shading; counts) and 850-hPa LLWs (vector; in meters per second) upon the WTNA index for the period 1980–2024; (**B**) same as (A) but for the North Atlantic; (**C**) regressed Northwest Pacific TCGF anomalies and LLWs upon the CTNA index; and (**D**) same as (C) but for the North Atlantic. The dots and black vectors represent areas where anomalies are statistically significant above the 90% confidence level based on the two-tailed Student’s *t* test.

We next examine the relationship between the two TNA flavors with TC genesis frequency (TCGF) across the two basins. Consistent with previous studies (*4*, *9*, *15*, *16*), the canonical TNA warming is associated with a significant suppression of Northwest Pacific TCGF (*r* = −0.46*, where * means *P* value < 0.1) and an enhancement of TCGF across the North Atlantic basin (*r* = 0.37*) over the 1980–2024 period. However, when the canonical TNA warming is decomposed into the WTNA and CTNA components, we find that the WTNA is the predominant driver of the suppressed Northwest Pacific TCGF (Fig. 2A; *r* = −0.37*), yet its influence on North Atlantic TCGF is weak and statistically insignificant (Fig. 2B; *r* = 0.18). In contrast, the CTNA suppresses TCGF in the eastern Northwest Pacific (Fig. 2C; *r* = −0.29*), thereby compensating the weak increase in the regressed TCGF anomaly in the region under the WTNA pattern (Fig. 2A). The North Atlantic TCGF anomaly associated with the CTNA explains the major portion of the North Atlantic TCGF variability in response to the TNA SST anomaly (Fig. 2D; *r* = 0.35*). The statistics of variance analysis (see the “Definitions of TNA, WTNA, and CTNA indices” section in Materials and Methods; fig. S4) (*29*, *30*) shows that the WTNA accounts for 64.7% (23.7%) of the canonical TNA impact on Northwest Pacific (North Atlantic) TCGF, while the CTNA explains 39.7% (89.5%). Hence, the canonical TNA warming impact on TCGF in the two basins is a superposition of the WTNA and CTNA influences, with a clear asymmetry that the CTNA exerts a dominant control over North Atlantic TCGF, whereas the WTNA has a stronger impact on Northwest Pacific TCGF.

### Mechanisms behind the distinct impacts 

**Fig. 3.**
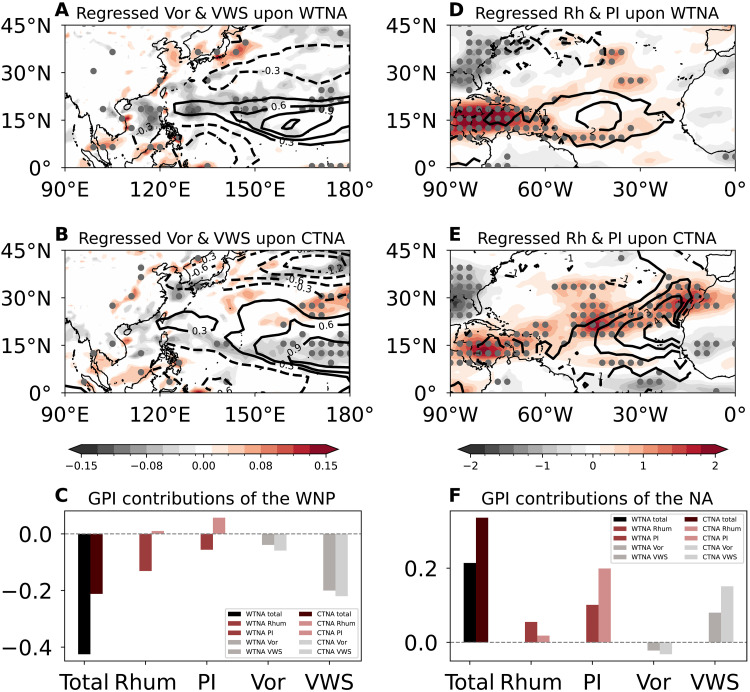
Impacts of the two TNA flavors on thermodynamic and dynamic factors and their relative contributions to the GPI. (**A**) Regressed relative vorticity (Vor) at 850 hPa (shading; 10^−5^ s^−1^) and vertical wind shear (VWS; contour; in meters per second) over the Northwest Pacific upon the normalized WTNA index for the period 1980–2024; (**B**) same as (A) but regressed onto the CTNA index; (**C**) regressed GPI anomalies over the Northwest Pacific (WNP) onto the WTNA (CTNA) index and their relative importance (bars) of individual factors; (**D**) regressed relative humidity (Rhum) at 600 hPa (shading; in percentage) and potential intensity (PI; contour; in meters per second) over the North Atlantic upon the WTNA index; (**E**) same as (D) but regressed onto the CTNA index; and (**F**) same as (C) but for the North Atlantic (NA). The indices in (C) and (F) are area-averaged over 130°E to 180°, 10° to 20°N for the Northwest Pacific and over 10°W to 60°W, 5° to 15°N for the North Atlantic. The dots represent areas where anomalies are statistically significant above the 90% confidence level based on the two-tailed Student’s *t* test.

To elucidate the physical mechanisms underpinning the pronounced asymmetric impacts of TNA flavors on TCGF in the North Atlantic and Northwest Pacific, we examine both dynamic and thermodynamic influences on TC genesis (figs. S5 to S7). Over the Northwest Pacific, the WTNA warming reduces low-level vorticity and enhances vertical wind shear over the South China Sea and east of the Philippines (Fig. 3A), thereby suppressing TC activity over the Northwest Pacific. In contrast, the CTNA warming produces positive vertical wind shear anomalies and negative vorticity anomalies over the eastern Northwest Pacific (Fig. 3B), aligning with the local suppression of TCGF shown in Fig. 2C. Thermodynamic conditions play a comparatively limited role. The 600-hPa relative humidity anomalies in response to TNA, WTNA, and CTNA warmings exhibit broadly similar patterns over the Northwest Pacific (fig. S5), indicating that this factor contributes little to the differentiated TCGF response in this basin. Consequently, the spatial pattern of TCGF suppression over the Northwest Pacific is primarily governed by dynamic controls. This dominance is further supported by the genesis potential index (GPI) analysis (see the “GPI and relative importance contributed by distinct modes” section in Materials and Methods; fig. S7), which shows the negative GPI anomalies primarily driven by enhanced vertical wind shear (Fig. 3C), whereas the thermodynamic contributions (e.g., relative humidity and potential intensity) are secondary.

Over the North Atlantic, the canonical TNA warming and both TNA flavors exhibit slightly negative low-level vorticity anomalies in the deep tropics (fig. S5, A to C), which is inconsistent with the observed increase in North Atlantic TCGF. Although vertical wind shear anomalies are broadly consistent with changes in the TCGF patterns, with a decrease over the MDR of the North Atlantic during warm phases of both flavors (fig. S6), their spatial differentiation between the WTNA and CTNA remains less pronounced. In contrast, the relative humidity and potential intensity anomalies exhibit notable differences between the WTNA and the CTNA (Fig. 3, D and E). During WTNA warming, the increased relative humidity is largely confined to the western Gulf of Mexico (Fig. 3D), and the positive potential intensity anomalies are restricted to the western-central TNA. By contrast, the CTNA warming induces an increase in potential intensity, and relative humidity is more extensively stretching from the Gulf to the West African coast (Fig. 3E), providing a more favorable environment for TC genesis. The conducive condition is supported by GPI analysis (Fig. 3F), indicating that the thermodynamic factors, particularly potential intensity, play a dominant role in enhancing TC genesis, while the contribution from dynamic factors is secondary. Mechanistically, the WTNA warming shows that SST anomaly is centered around 60°W (Fig. 1D), superimposing onto the Atlantic warm pool, effectively triggering a low-level cyclonic Rossby response over the Gulf of Mexico. This westward-shifted potential intensity and relative humidity anomaly are less efficient for TCGF over the North Atlantic. However, the CTNA warming weakens the zonal SST gradient and trade winds (Fig. 1E), leading to a more spatially coherent increase in potential intensity and relative humidity over the North Atlantic (Fig. 3E), thereby favoring enhanced TC genesis.

To test the above mechanisms, we perform a set of sensitivity experiments based on a high-resolution atmospheric model (HiRAM), which is capable of resolving TC-like vortices (see the “HiRAM experiments” section in Materials and Methods; figs. S8 to S10) (*31*–*33*). The control run is forced by monthly climatological SST averaged from 1980 to 2024 and is parallelly integrated 1 year to generate 30 ensemble members with their initial conditions being slightly perturbed. The TNA run is the same as the control run but with the regressed TNA SST anomalies during the TC season are superimposed onto the climatological SSTs. The WTNA and CTNA runs are similar to the TNA run but with the warm pool (Fig. 1D) and coastal (Fig. 1E) SST anomalies added, respectively.

The canonical TNA warming generally imposes broad impacts on basin-wide low-level circulations over both the North Atlantic and Northwest Pacific (fig. S8A). However, the WTNA warming–induced cyclonic circulation anomalies are confined over the Atlantic warm pool region with a weak and insignificant impact in the eastern North Atlantic (fig. S8B), which is less efficient to promote TC genesis. Meanwhile, there is still an anticyclonic circulation anomaly over the Northwest Pacific in response to the WTNA warming (fig. S8B), consistent with the canonical TNA warming impact on TC genesis in this basin.

The CTNA warming triggers strong westerly wind anomalies with a cyclonic shear in the North Atlantic, conducive to TC genesis locally (fig. S8C). However, the weakened low-level easterly wind anomalies in response to the CTNA warming are confined to the eastern Northwest Pacific (fig. S8C). Moreover, the upper-level winds show a large difference in equatorial easterly anomalies between the WTNA and CTNA runs over the Indian Ocean (fig. S9, B and C). The difference accords with the observations that the WTNA warming excites upper-level Kelvin waves and affects the Northwest Pacific through the relay of the Indian Ocean (Fig. 1, D versus E, and figs. S3, B versus C, and S9, B versus C) (*15*, *17*, *18*). Both observed and simulated results suggest that the suppression of Northwest Pacific TCGF is primarily driven by dynamically induced changes in vertical wind shear and low-level vorticity associated with both the WTNA and CTNA warmings, while the North Atlantic TCGF is effectively enhanced by the CTNA warming–induced increases in potential intensity and mid-level relative humidity in the North Atlantic.

### Quantification of contributions via high-resolution simulations

**Fig. 4.**
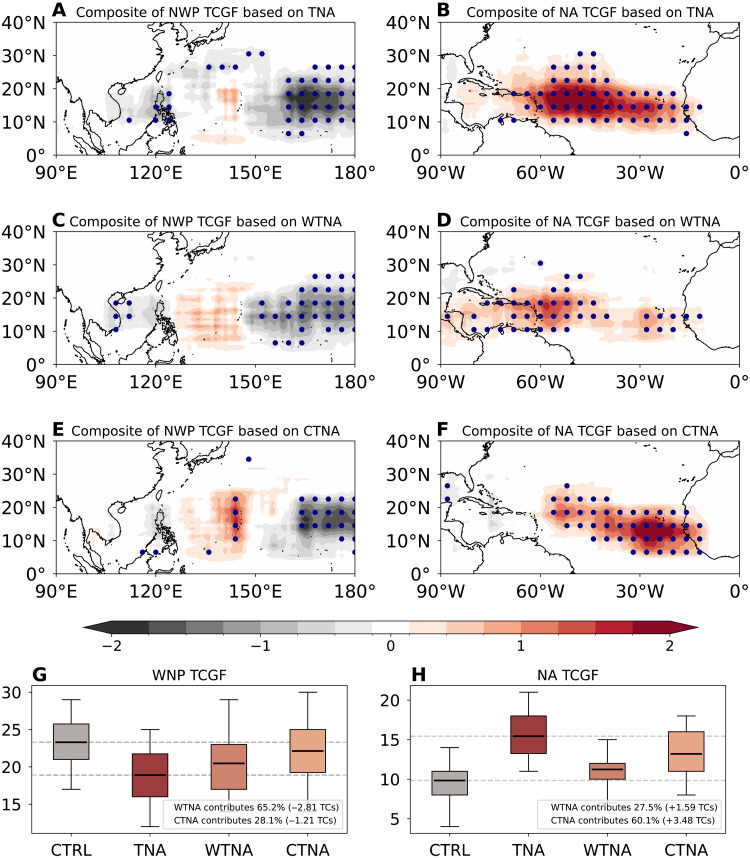
Seasonal TCGF anomalies and their quantification associated with the different TNA forcing experiments. (**A**) Northwest Pacific (NWP) TCGF difference between the canonical TNA and CTRL runs; (**B**) same as (A) but for the North Atlantic (NA); (**C**) Northwest Pacific TCGF difference between the WTNA and CTRL runs; (**D**) same as (C) but for the North Atlantic; (**E**) Northwest Pacific TCGF difference between CTNA and CTRL runs; (**F**) same as (E) but for the North Atlantic; (**G**) simulated Northwest Pacific TCGF in CTRL, TNA, WTNA, and CTNA runs; and (**H**) same as (G) but for the North Atlantic. The dots in (A) to (F) represent areas where anomalies are statistically significant above the 90% confidence level based on the two-tailed Student’s *t* test. In (G) and (H), the box-and-whisker plots show the distribution of simulated TCGF among 30 ensemble members, with whiskers indicating ±1.5 SDs, boxes denoting the 25th to 75th percentiles, and horizontal solid lines representing the median. Two horizontal dashed lines represent the medians of simulated TCGFs in the CTRL and TNA runs.

We further examine the simulated impacts of TNA flavors on TCGF changes in the North Atlantic and Northwest Pacific based on the sensitivity numerical experiments discussed above. Simulated TCGF changes relative to the control run (Fig. 4) show that the canonical TNA warming reproduces the suppression of TCGF in the Northwest Pacific (Figs. 4A versus 2A) and enhancement in the North Atlantic (Figs. 4B versus 2B). The WTNA warming captures the overall decrease in TCGF in the Northwest Pacific despite a slight increase in the east of Philippines (Fig. 4C), but its impact on North Atlantic TCGF is weaker than that of canonical TNA warming (Fig. 4, D versus B), indicating that the WTNA warming dominates the influence on Northwest Pacific TCGF while its impact on North Atlantic TCGF is moderate.

In contrast, the CTNA warming shows a limited suppression of TCGF over the eastern Northwest Pacific (Fig. 4E) but a substantial enhancement of TCGF across the North Atlantic (Fig. 4, F versus D). We further quantify the contributions of the canonical TNA and its two distinct flavors to TCGF changes (Fig. 4, G and H). Relative to the control run (23.45 TCs), the TNA run yields a reduced mean TCGF of 19.14, corresponding to a suppression of 4.31 TCs over the Northwest Pacific in response to 2 SDs of TNA SST anomalies (see the “HiRAM experiments” section in Materials and Methods). Among the total reduction, the WTNA and CTNA warmings account for around 65.2% (−2.81 TCs) and 28.1% (−1.21 TCs), respectively (Fig. 4G). Over the North Atlantic, the ensemble-mean TCGF increases from about 9.69 TCs in the control run to 15.48 TCs in the TNA run, with the CTNA warming accounting for 60.1% and WTNA warming for 27.5% (Fig. 4H). These results, consistent with the observational analysis, confirm that the WTNA warming exerts a dominant influence on TCGF suppression over the Northwest Pacific, while the CTNA warming plays a primary role in enhancing TCGF over the North Atlantic.

### Projected changes of TNA flavors under global warming

**Fig. 5.**
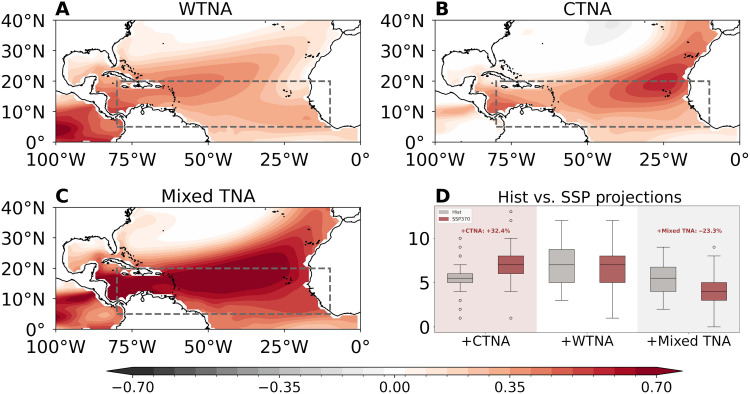
Clustering analysis of TNA flavors and their future changes. (**A**) WTNA pattern extracted from clustering analysis based on large ensemble simulations; (**B**) CTNA pattern; (**C**) mixed-type TNA pattern characterized by overlapped WTNA and CTNA with stronger magnitudes; and (**D**) box-and-whisker plots showing the frequency of positive TNA events in historical (gray) and SSP370 (red) simulations. The box-and-whisker plots show the projected CTNA, WTNA, and mixed-type TNA events among 50 ensemble members, with whiskers indicating ±1.5 SDs, boxes denoting the 25th to 75th percentiles, and horizontal lines representing the median. The circles in (D) represent the projected number of TNA events above ±1.5 STD across 50 ensemble members.

Given the diverse impacts of two TNA flavors on TCGF over the Northwest Pacific and North Atlantic, we examine their future changes and anticipated impacts on TC activity. Observational records show a notable shift over the past seven decades (1950–2024). When comparing two periods (1950–1987 and 1988–2024), the occurrence of warm CTNA events increased from 18.2% (6 cases) to 34.5% (10 cases), while the warm WTNA events decreased from 24.3% (8 cases) to 13.8% (4 cases). The frequency of negative mixed-type TNA events also decreased from 12.1 to 6.9%, although case numbers remained small (Fig. 5). Other phases of these types exhibit minor changes. The results thus suggest an increase in the CTNA warming events and increased impacts on North Atlantic TCGF. Previous studies have documented that the sea surface in the TNA warms faster than in the tropical Pacific in the past half century since 1960 (*6*, *34*, *35*), which has contributed to intensified interannual SST variability in the former. Moreover, the mean state warming is conducive to CTNA variability due to the relatively low SST compared with the WTNA (*36*), resembling the Pacific basin (*37*, *38*).

To assess future changes, we compare the occurrences of the two TNA flavors based on 50-member large-ensemble simulations for both the historical simulations and projections under the Shared Socioeconomic Pathway 3-7.0 scenario (SSP370; Fig. 5; see the “Large ensemble data” section in Materials and Methods). Here, TNA events with amplitude exceeding ±0.8 SD are classified into three groups: WTNA (Fig. 5A), CTNA (Fig. 5B), and mixed-type groups (Fig. 5C), using a clustering analysis (see the “Clustering analysis” section in Materials and Methods). A total of 1865 TNA events is identified in historical simulations among the 4300 model years, accounting for 43.37% of all years. Consistent with observations, the frequency of positive CTNA events increase markedly, from 281 cases (5.62 averaged in a single member) to 372 cases (7.44), accounting for 32.4% increase relative to historical simulations (Fig. 5D). In contrast, the positive mixed-type events decreased by 23.3%, from an average of 5.5 events in historical simulations to 4.22 in the SSP370 simulations (Fig. 5D). The frequency of positive WTNA events and all negative events shows little change under the two simulations. The results indicate a shift toward more frequent positive (warm) CTNA events under a warmer climate. Given the dominant role of CTNA warming in enhancing North Atlantic TCGF, the projection suggests an intensification of CTNA-driven impacts on Atlantic TC activity under continuing global warming.

## DISCUSSION

We find that the TNA SST warm anomalies have distinct impacts on TCGF over the North Atlantic and Northwest Pacific from the two TNA flavors, referred to as the WTNA and the CTNA (Fig. 1). The WTNA warming dominates the decrease of TCGF over the Northwest Pacific, while its impact on North Atlantic TCGF is secondary compared with the CTNA warming (Figs. 2 and 4). The CTNA warming dominates the increase of North Atlantic TCGF, whereas its influence on Northwest Pacific TCGF is limited to the eastern Northwest Pacific. The dynamic factors including vertical wind shear and low-level vorticity contribute to the asymmetric impact of the WTNA on Northwest Pacific TCGF; by contrast, the thermodynamic factors including potential intensity and mid-level relative humidity dominate the skewed influence of the CTNA on North Atlantic TCGF. The WTNA warming accounts for 65.2% of the suppression in Northwest Pacific TCGF, while the CTNA warming drives a 60.1% increase of North Atlantic TCGF, both relative to the canonical TNA. Large ensemble simulations project a 32.4% increase in the frequency of positive CTNA events by the end of the 21st century, suggesting a substantial intensification of North Atlantic TC activity under greenhouse warming. Together, these results highlight CTNA variability as an increasingly important driver of North Atlantic TC activity in a warmer climate.

The canonical TNA warming in spring may trigger ENSO events in the following winter (*4*, *5*), and previous studies have also found a stationary correlation between the WTNA and ENSO (*20*). However, the connection between the CTNA and ENSO has been weak and statistically insignificant since the 1990s, suggesting an increased independence of the CTNA with ENSO (*20*). This emerging cross-basin decoupling highlights a potential of the CTNA as an independent source of predictability and underscores the importance of its future changes and potential impacts on North Atlantic TC activity.

## MATERIALS AND METHODS

### Best-track TC and reanalysis data

We use the TC best-track data from the International Best Track Archive for Climate Stewardship (*39*, *40*) for 1980–2024, which contains 6-hourly TC center (longitude and latitude) and intensity (maximum-sustained surface wind) information. The TC genesis location is defined when its surface wind speed first reached or exceeded 35 knots (about 17.2 m s^−1^) during its life span. The Northwest Pacific and North Atlantic TC season is defined as the months from June to November.

We use the monthly atmospheric data, including 850- and 200-hPa winds, low-level relative vorticity at 850 hPa, mid-level relative humidity at 600 hPa, and vertical zonal wind shear between 200 and 850 hPa from the fifth generation of European Centre for Medium-Range Weather Forecast reanalysis (*41*) with all variables interpolated into 2.5° × 2.5° mesh grids. The monthly SST data are derived from the Hadley Centre Sea Ice and Sea Surface Temperature (*42*) with a horizontal resolution of 1° × 1°. The monthly precipitation data are from the global precipitation climatology project (*43*) covering the period from 1980 to present.

### Large ensemble data

Outputs of large ensemble simulations with two scenarios are used in this study, which are downloaded from the Community Earth System Model (CESM), version 2. Both the historical and SSP370 simulations, a medium greenhouse gas emission scenario, contain 50 ensemble members that differ with the initial conditions. The historical runs cover the period from 1850 to 2014, and the SSP370 contains the integration from 2015 to 2100 forced by a medium greenhouse gas emission in the fully coupled model. We use the SST data to derive the TNA flavors based on clustering method as introduced below. Here, we use the CESM to derive the TNA warming flavors as this model is particularly suitable for investigating the cross-basin interactions among climate modes (*4*, *8*, *44*, *45*), including the TNA. However, previous studies have indicated that CESM could underestimate the climatological frequency of TC genesis over the North Atlantic region (*46*, *47*). Therefore, we rely on the HiRAM from the Geophysical Fluid Dynamics Laboratory (GFDL) for the numerical experiments of TC activity while using CESM to derive the future changes of TNA warming flavors.

### Definitions of TNA, WTNA, and CTNA indices

The canonical TNA index is defined as the SST anomaly averaged over the area of 5°N to 20°N, 10°W to 80°W (Fig. 1, B and C, and fig. S1). We further derive the two TNA flavors based on a 45° rotated empirical orthogonal function analysis following steps below: First, we conduct the empirical orthogonal function analysis of the detrended June–November (JJASON) SST from 1980 to 2024 and extract two spatial patterns as shown in fig. S2 (A and C). The first mode contains both the WTNA and CTNA modes, while the second mode captures the SST concentrated in the coastal region of West Africa (fig. S2, A and C). These two separated modes are mathematically orthogonal. We therefore construct the WTNA and the CTNA indices through rotating the two principal components (PC1 and PC2) based on empirical orthogonal function analysis (*48*–*50*)

$$\text{WTNA}=\frac{\left(\mathrm{PC}1-\mathrm{PC}2\right)}{\sqrt{2}}$$ (1)

$$\text{CTNA}=\frac{\left(\mathrm{PC}1+\mathrm{PC}2\right)}{\sqrt{2}}$$ (2)

where the WTNA represents the warm-pool type, while the CTNA stands for the coastal type. Equations 1 and 2 indicate that we counter-rotate the original principal components by 45°, hence the WTNA and the CTNA modes are orthogonal with their SST spatial patterns reflecting the realistic coastal and warm-pool patterns (fig. S2, B and D). The original and rotated principal components are shown in fig. S2 (E and F). It is worth noting that the PC1 for both 2023 and 2024 is very strong (fig. S2E), reflecting the warm anomalous states during the 2 years. However, the PC2 is opposite, represented by respective red and blue dots in 2023 and 2024 (fig. S2E). The coastal cooling in 2024 is totally overlooked if we only focus on the warm anomaly rather than the nonuniform spatial distribution. After the rotation, both PC1 and PC2 are positive in 2023, indicating a combination of positive WTNA and CTNA events (fig. S2F). However, it shows a strong WTNA warming in 2024, while the CTNA is in a weak negative phase for the June to November season.

The correlation coefficient between Northwest Pacific (North Atlantic) TCGF and canonical TNA index is −0.46 (0.37), both above the 90% confidence level based on the two-sided Student’s *t* test. The correlation coefficient between North Atlantic TCGF and WTNA index is 0.18 (*P* value > 0.1), while the correlation between North Atlantic TCGF and CTNA index is 0.35 (*P* value < 0.1). The correlation coefficients are both significant (*r* = −0.37 and *r* = −0.29) at the 90% confidence level between Northwest Pacific TCGF and WTNA (CTNA) index. Note that all correlation coefficients are calculated on the basis of detrended time series. In terms of the variance change (*29*, *30*), the WTNA mode accounts for 64.7% of the canonical TNA-induced Northwest Pacific TCGF variability, while the CTNA mode contributes 39.7% with a relatively medium spread (fig. S4A). However, the WTNA mode contributes 23.7% to North Atlantic TCGF variability induced by the canonical TNA, while the CTNA mode exerts a predominant impact (89.5%; fig. S4B).

### GPI and relative importance contributed by distinct modes

The GPI proposed by Emanuel and Nolan (*51*) is used to evaluate TC genesis and relative importance of dynamic and thermodynamic factors in low-resolution models and reanalysis. The GPI is defined as

$$\text{GPI}={|10^{5}\eta |}^{3/2}{\left(\frac{\text{RH}}{50}\right)}^{3}{\left(\frac{\text{PI}}{70}\right)}^{3}{\left(1+0.1\text{VWS}\right)}^{-2}$$ (3)

where η is the combination of relative vorticity and Coriolis parameter at 850 hPa, RH is the relative humidity at 600 hPa, PI represents the potential intensity of a TC that can reach theoretically, and VWS indicates the vertical wind shear between 200 and 850 hPa. We evaluate the relative importance of the above four factors by varying one factor with others fixed to the climatology based on previous studies (*52*, *53*).

### HiRAM experiments

We further conduct sensitivity experiments based on the HiRAM (*29*, *33*, *54*, *55*) from the GFDL, a cubic-sphere model (*56*) with a horizontal resolution of ~50 km (TC permitting) (*29*, *55*) and 32 vertical levels. The experiments contain a control run (CTRL) and three sensitivity runs. The control run is forced by the monthly climatological SST between 1980 and 2024, integrated from 1st January to 31st December. Thirty ensemble members are generated by randomly perturbing the initial conditions. The TNA run is identical to the CTRL except that we add regressed SST anomalies (fig. S1A) upon the canonical TNA index from June to November. The WTNA (CTNA) run is the same as the TNA run except that we add regressed SST anomalies upon the WTNA (CTNA) index to the control run (Fig. 1, D and E). Given the magnitude of a strong WTNA or CTNA event can be greater than 2 to 3 SDs (Fig. 1, B and C), we add SST anomalies in response to 2 SDs of indices. The differences among the three sensitivity runs with the CTRL run represent the combined or isolated impacts of the canonical TNA and two TNA flavors on TCGF (Fig. 4) and associated atmospheric circulations (figs. S8 and S9). Both observed and simulated Northwest Pacific TCs are counted over 0° to 45°N, 100°E to 180°, and North Atlantic TCs are counted from the Gulf of Mexico to the West African coast, 0° to 45°N. The high-resolution model outputs reproduce the spatial distribution of climatological TC tracks and geneses (fig. S10).

### Spatial interpolation of TCGF

Following previous studies (*53*), the TCGF on each grid box is counted as the number of TCs generated in an area of 20° in the zonal direction and 10° in the meridional direction away from the center of the grid box, and the grid spacing is 1° by 1°. This method not only offsets the uncertainty and deviation of TC genesis locations but also establishes a stable relationship between TCGF and related environmental factors such as the TNA. We choose a 20° by 10° box because the synoptic waves (such as equatorial Rossby waves, mixed Rossby–gravity waves, and easterly waves or other types of synoptic disturbances) that trigger TC genesis have a zonal scale of about 2000 km and a meridional e-folded scale.

### TC tracker and model validation

Following the TC detection algorithm developed by the GFDL (https://gfdl.noaa.gov/tstorms/) (*33*, *57*, *58*), we explicitly extract TC information from the HiRAM. The extraction criteria are as follows: First, a low-pressure center is detected on the sea surface. Considering that the TC intensity is initially weak and the structure is asymmetrical, the surface isobars are not necessary to be closed. Second, for the vortex to reach a TC strength, the surface wind speed is not less than 17.5 m s^−1^ for the 0.5° model for at least once during their life spans, and the absolute value of relative vorticity at 850 hPa is greater than 5 × 10^−4^ s^−1^. Third, the temperature within the range of 1200 km of the detected TC center is higher than the temperature between 1200 and 2400 km from the TC center at 300 to 500 hPa to ensure a warm core structure. Last, the TC life span should have been at least 3 days. The climatological TC tracks and genesis locations in the four experiments are shown in fig. S10.

### Clustering analysis

The *k*-means clustering analysis (*59*, *60*) is applied to classify TNA events into clusters. It is a centroid-based clustering method using squared Euclidean distance to measure the “similarity” between members. Here, we select TNA events based on 0.8 SD from large ensemble datasets of the CESM, version 2. We reverted negative TNA events into positive ones by multiplying −1 to avoid separating positive and negative events into different clusters. We do not use multiple climate models since the clustering method is sensitive to different sample sizes across models. We use large ensemble simulations from the CESM because the WTNA and CTNA are well reproduced in the historical simulations. We also find poor simulations of the WTNA and CTNA in other large ensembles, such as those from the Max Planck Institute (*61*) and Canadian Earth System (*62*) models. We test the cluster number (*k*) from 2 to 4 and find that 3 is the best to represent the clusters (Fig. 5), since it separates the TNA events into the WTNA, CTNA, and mixed-type TNA. We use the periods from 1929 to 2014 in the historical run and from 2015 to 2100 in the SSP370 run, 86 years for each member. We select 1865 TNA cases (positive and negative events) in the historical run and 1826 cases in the SSP370 run. Frequency of positive CTNA events increases from 281 in historical run to 372 in SSP370 run, consistent with the observed increase.
