# Supplementary material for: Distinct impacts of tropical North Atlantic warming flavors on cross-basin tropical cyclone activity
Source: Sci Adv. 2026 May 20;12(21):eaeb2316. doi: 10.1126/sciadv.aeb2316 (PMC13189095; doi:10.1126/sciadv.aeb2316)
Supplement: Supplementary file 1 — Figs. S1 to S10 [file sciadv.aeb2316_sm.pdf]

Science Advances
AAAS

Supplementary Materials for

Distinct impacts of tropical North Atlantic warming flavors on cross-basin tropical cyclone activity

Jiuwei Zhao *et al.*

Corresponding author: Ruifen Zhan, zhanrf@fudan.edu.cn; Yi Liu, ly6161yl@gmail.com

Sci. Adv. **12**, eaeb2316 (2026)
DOI: 10.1126/sciadv.aeb2316

This PDF file includes:

Figs. S1 to S10

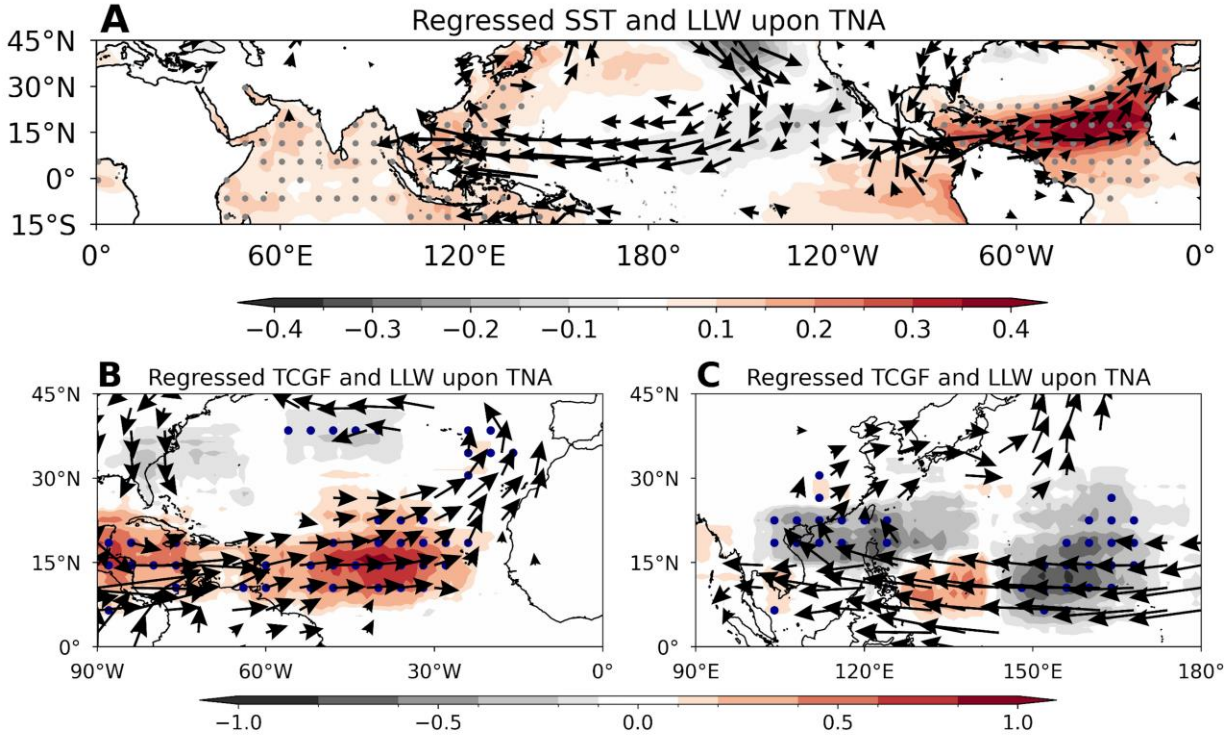

Fig. S1.

Regressed anomalies of sea surface temperature (SST), low-level winds (LLWs) and TCGF upon the canonical TNA index. (A) June-November (JJASON) averaged SST (°C) and LLW at 850 hPa regressed upon the canonical TNA index; (B) regressed anomalies of TCGF (shaded) and LLW (vector; m s^{-1}) over the North Atlantic; and **(C)** same as **(B)** but over the Northwest Pacific. The dots and black vectors represent areas where anomalies are statistically significant above the 90% confidence level based on a two-sided Student's t test.

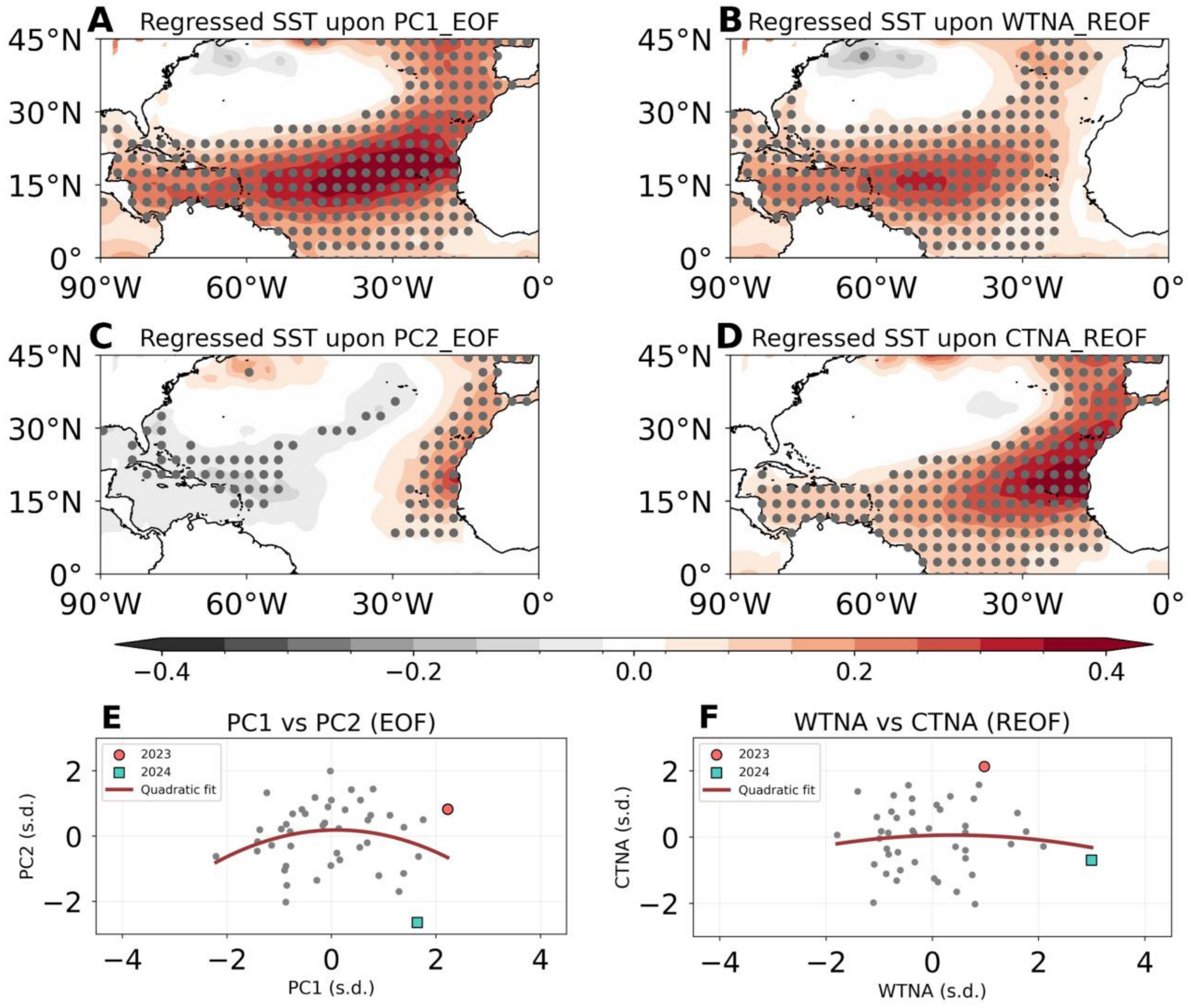

Fig. S2.

The empirical orthogonal function (EOF) analysis and their rotated results (REOF). (A) The first EOF mode of detrended June-November (JJASON) averaged SST anomalies from 1980 to 2024; **(B)** the WTNA mode derived from the rotated EOF analysis by summarizing two principal components (PC1-PC2)/ $\sqrt{2}$; **(C)** same as **(A)** but for the second EOF mode; **(D)** the CTNA mode based on the rotated EOF through the minus of the two (PC1+PC2)/ $\sqrt{2}$; **(E)** the scatter plots of original PC1 and PC2; and **(F)** the scatter plots of rotated principal components (WTNA and CTNA). The dots in (**A-D**) represent areas where anomalies are statistically significant above the 90% confidence level based on a two-sided Student's t test. The red and blue dots represent 2023 and 2024 in **(E)** and **(F)**.

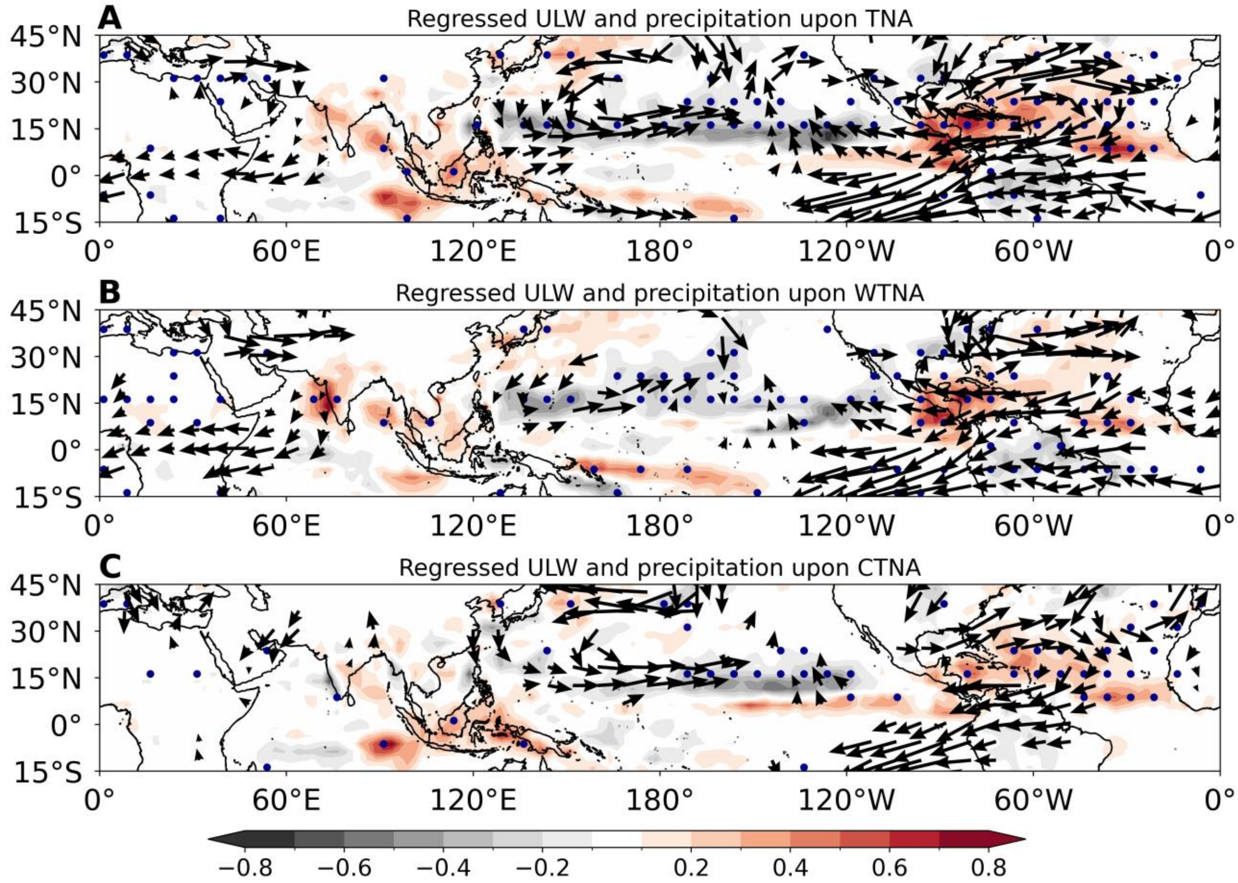

Fig. S3.

Regressed anomalies of precipitation (Pre) and upper-level winds (ULW) upon tropical North Atlantic indices. (A) Regressed Pre (shading; mm) and ULW at 200 hPa (vector; m s^{-1}) upon normalized TNA index for 1980-2024 period; **(B)** same as **(A)** but for the WTNA index; and **(C)** same as **(A)** but for CTNA. The dots and black vectors represent areas where anomalies are statistically significant above the 90% confidence level based on a two-sided Student’s t test.

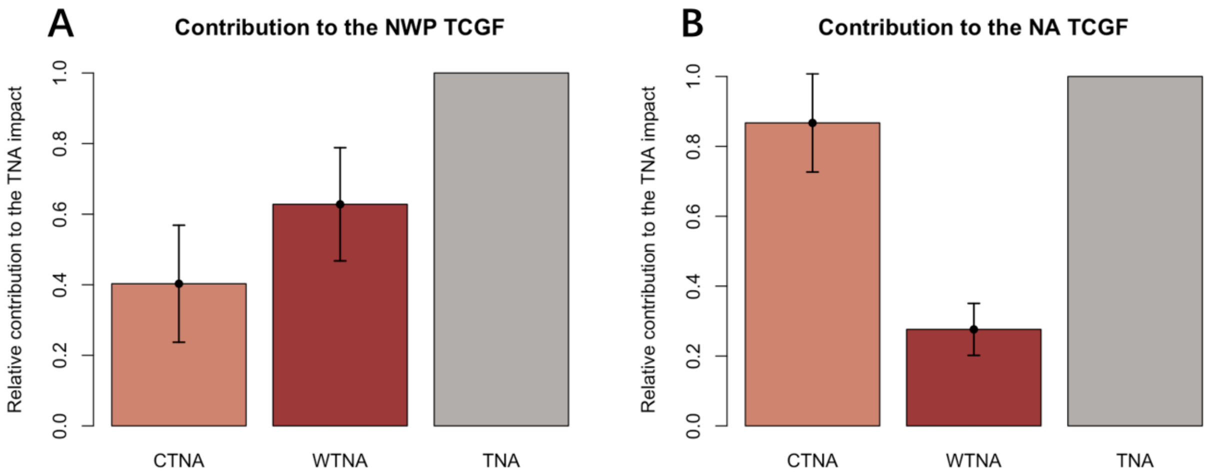

Fig. S4.

Relative importance of CTNA and WTNA to the TCGF change over the Northwest Pacific and North Atlantic compared with the TNA impact. (A) Relative contributions of CTNA and WTNA to Northwest Pacific TCGF change compared with the TNA impact; **(B)** same as (A) but for the North Atlantic TCGF variability. Here, we normalized the importance of TNA impact on TCGF variability to 1.

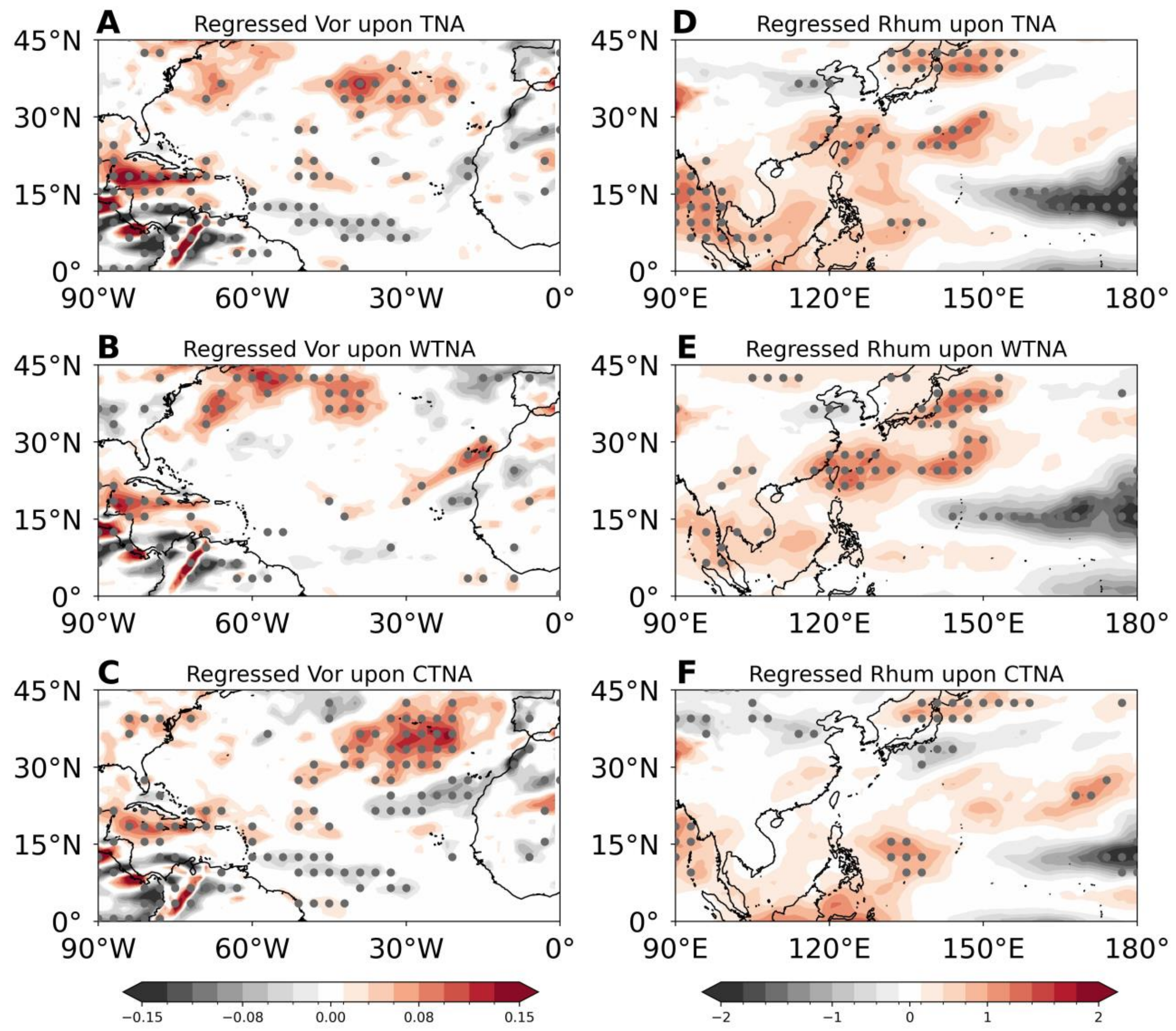

Fig. S5.

Regressed low-level relative vorticity (Vor) and mid-level relative humidity (Rhum) upon tropical North Atlantic (TNA) indices. (A) Regressed relative vorticity at 850hPa (shading; 10^{-5} s^{-1}) over the North Atlantic upon normalized TNA index for 1980-2024 period; **(B)** same as **(A)** but regressed upon the WTNA index; **(C)** same as **(A)** but regressed upon the CTNA index; **(D)** regressed relative humidity at 600 hPa (shaded; %) upon the traditional TNA index in the NA; **(E)** same as **(D)** but regressed upon the WTNA index; and **(F)** same as **(D)** but regressed upon the CTNA. The dots represent areas where anomalies are statistically significant above the 90% confidence level based on a two-sided Student's t test.

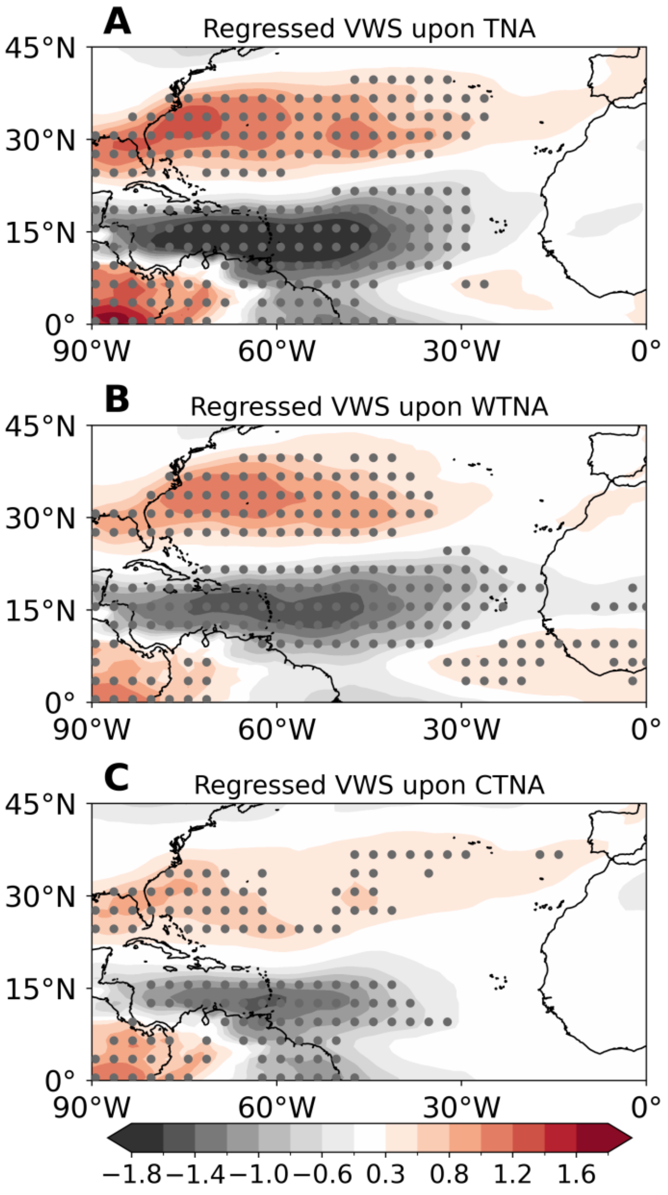

Fig. S6.

Regressed vertical wind shear (VWS) anomalies between 200 hPa and 850 hPa over the North Atlantic upon tropical North Atlantic (TNA) indices. (A) VWS anomalies (shading; m s^{-1}) regressed upon normalized TNA index for 1980-2024 period; **(B)** same as **(A)** but regressed upon the WTNA index; **(C)** same as **(A)** but regressed upon the CTNA index. The dots represent areas where anomalies are statistically significant above the 90% confidence level based on a two-sided Student's t test.

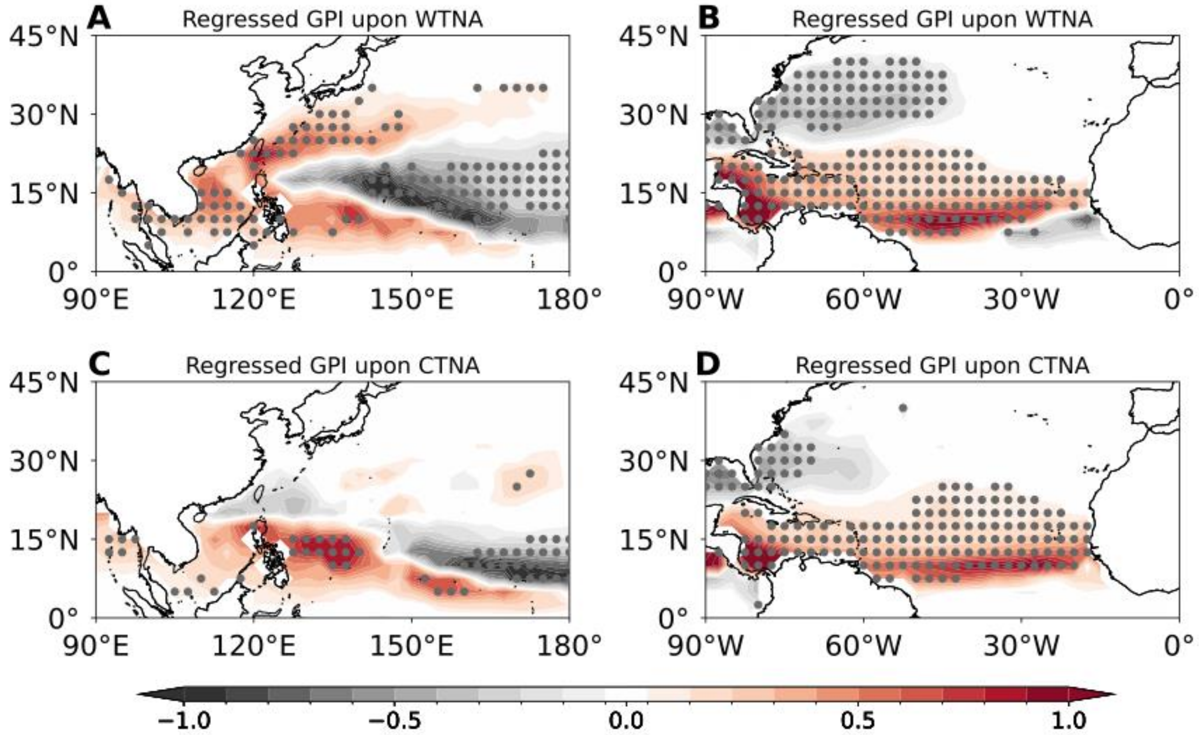

Fig. S7.

Regressed Genesis Potential Index (GPI) anomalies upon the TNA flavors. (A) GPI anomalies regressed upon the WTNA over the Northwest Pacific; **(B)** same as **(A)**, but for the North Atlantic; **(C)** GPI anomalies regressed onto the CTNA in the Northwest Pacific; and **(D)** same as **(C)**, but for the North Atlantic. The dots represent areas where anomalies are statistically significant above the 90% confidence level based on a two-tailed Student's t test.

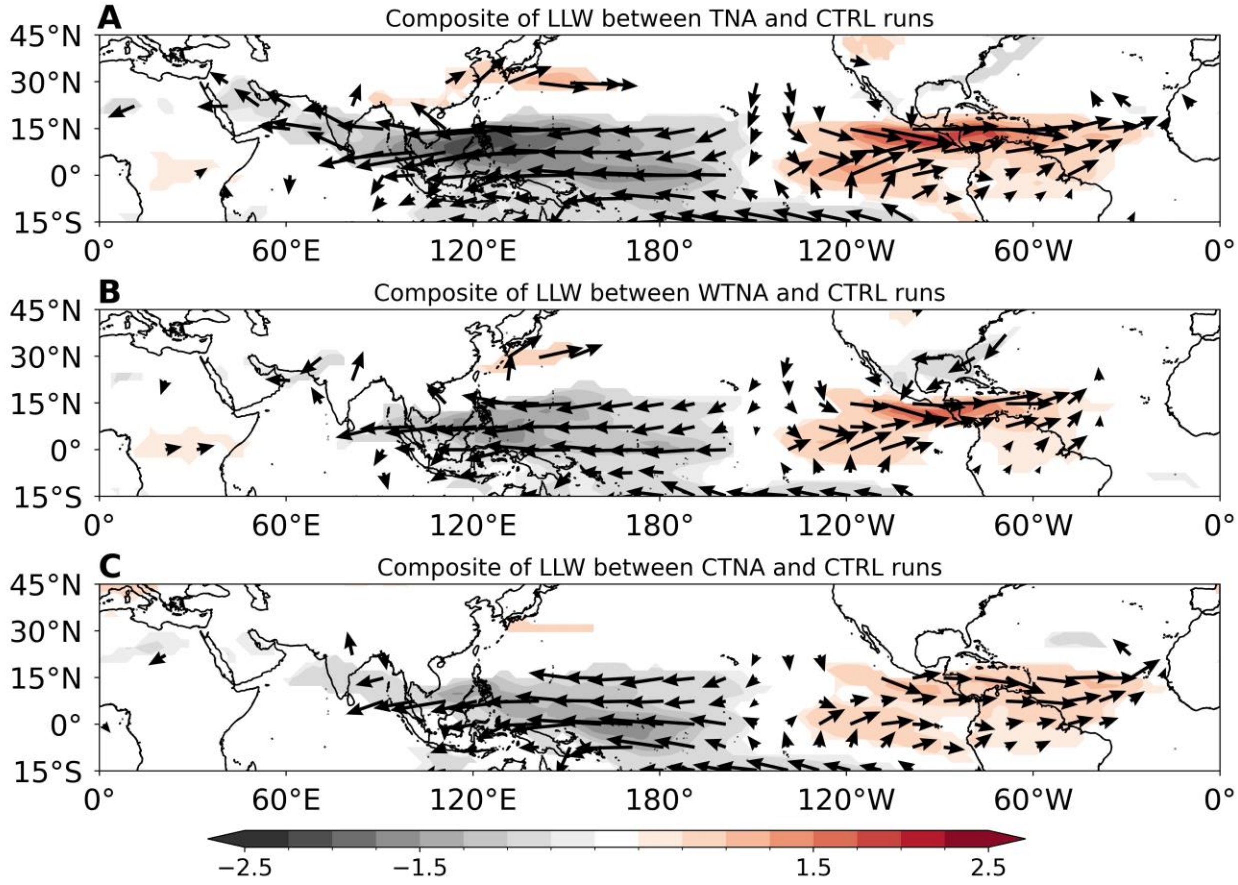

Fig. S8.

Low-level winds (LLW) difference between sensitive and control runs. (A) The June-November mean LLW anomalies at 850 hPa between the TNA run and control run based on the 30-member average; **(B)** the LLW anomalies between WTNA and control run; and **(C)** the LLW anomalies between CTNA and control run. The shading represents area above 95% confidence level for zonal wind based on a two-tailed Student’s t test.

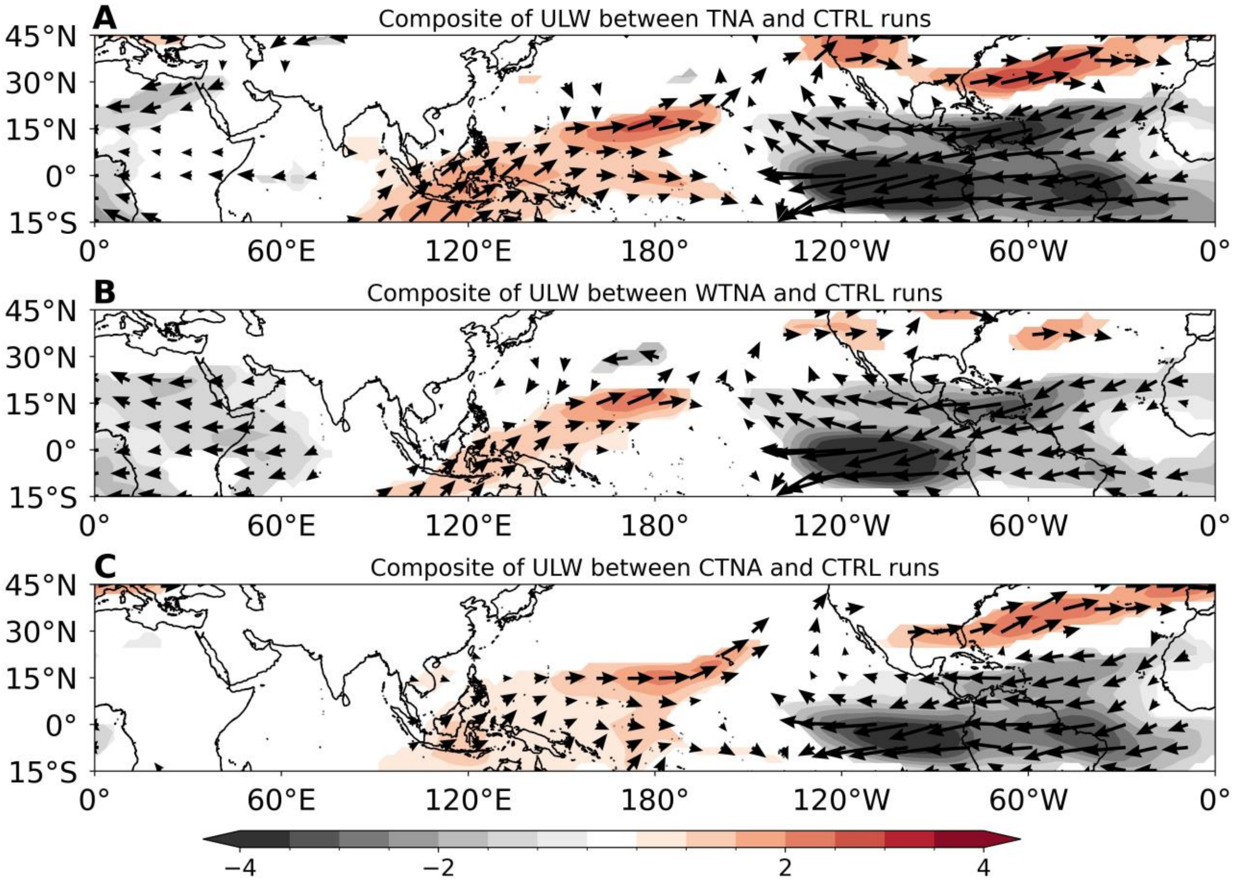

Fig. S9.

Upper-level winds (ULW) differences between sensitive and control runs. **(A)** The June-November mean ULW anomalies at 200 hPa between the TNA run and control run based on the 30-member average; **(B)** the ULW anomalies between WTNA and control run; and **(C)** the ULW anomalies between CTNA and control run. The shading represents area above 95% confidence level for zonal wind based on a two-tailed Student's t test.

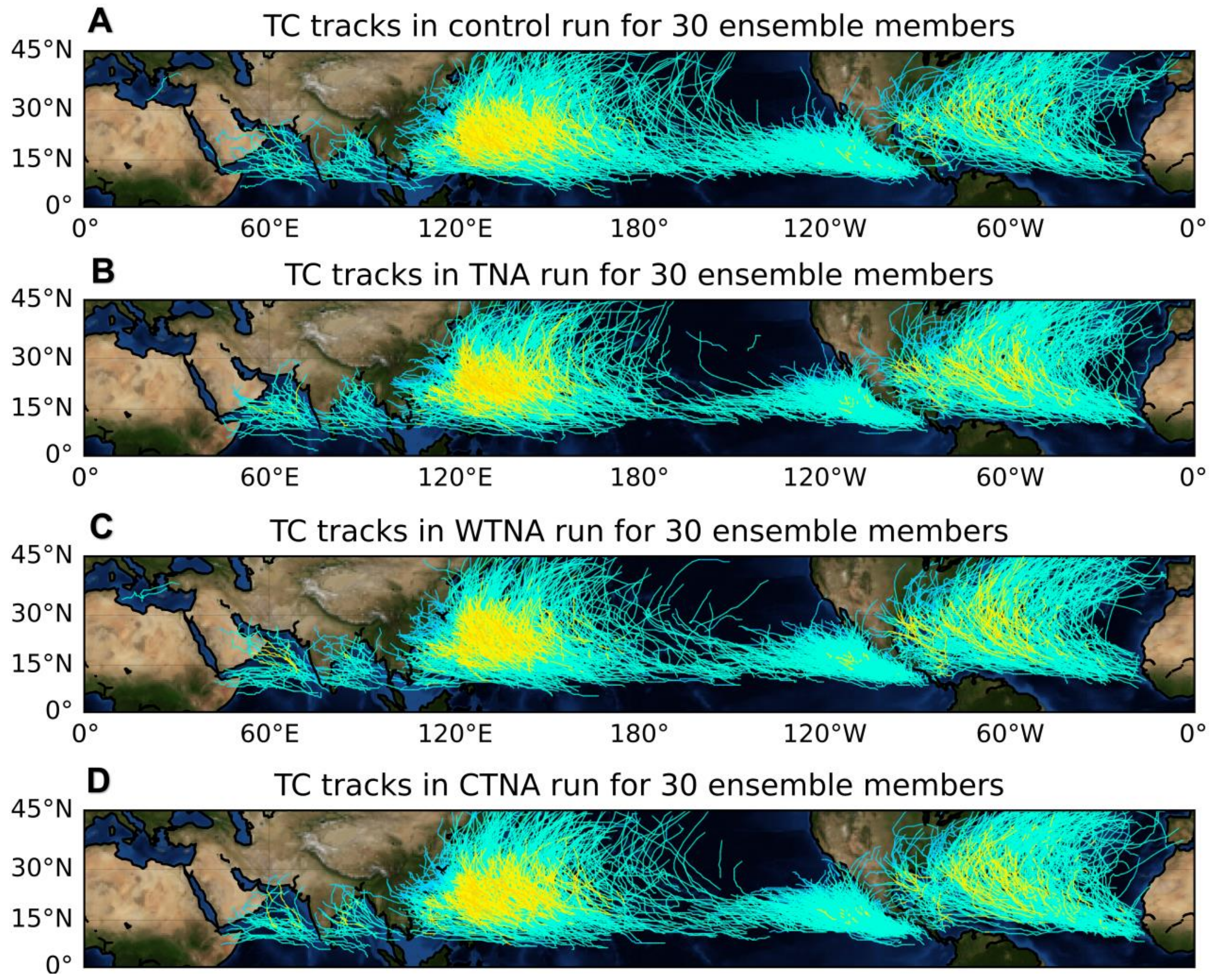

Fig. S10.

Distributions of TC tracks across 30 ensemble members in different experiments. (A) Control run; (B) TNA run; (C) WTNA run; and (D) CTNA run.
